# A novel approach to sonographic examination in a patient with a calf muscle tear: a case report

**DOI:** 10.4076/1752-1947-3-7291

**Published:** 2009-06-25

**Authors:** Carl PC Chen, Simon FT Tang, Chih-Chin Hsu, Ruo Li Chen, Rex CH Hsu, Chin-Wen Wu, Max JL Chen

**Affiliations:** 1 Department of Physical Medicine & Rehabilitation, Chang Gung Memorial Hospital and College of Medicine, Chang Gung University, Tao-Yuan County 333, Taiwan; 2 Pharmaceutical Sciences Research Division, King's College, Hodgkin Building, Guy's Campus, London SE1 1UL, UK; 3 Department of Medicine, Chang Gung University, College of Medicine, Tao-Yuan County 333, Taiwan

## Abstract

**Introduction:**

Rupture of the distal musculotendinous junction of the medial head of the gastrocnemius, also known as "tennis leg", can be readily examined using a soft tissue ultrasound. Loss of muscle fiber continuity and the occurrence of bloody fluid accumulation can be observed using ultrasound with the patient in the prone position; however, some cases may have normal ultrasound findings in this conventional position. We report a case of a middle-aged man with tennis leg. Ultrasound examination had normal findings during the first two attempts. During the third attempt, with the patient's calf muscles examined in an unconventional knee flexed position, sonographic findings resembling tennis leg were detected.

**Case presentation:**

A 60-year-old man in good health visited our rehabilitation clinic complaining of left calf muscle pain. On suspicion of a ruptured left medial head gastrocnemius muscle, a soft tissue ultrasound examination was performed. An ultrasound examination revealed symmetrical findings of bilateral calf muscles without evidence of muscle rupture. A roentgenogram of the left lower limb did not reveal any bony lesions. An ultrasound examination one week later also revealed negative sonographic findings. However, he still complained of persistent pain in his left calf area. A different ultrasound examination approach was then performed with the patient lying in the supine position with his knee flexed at 90 degrees. The transducer was then placed pointing upwards to examine the muscles and well-defined anechoic fluid collections with areas of hypoechoic surroundings were observed.

**Conclusion:**

For patients suffering from calf muscle area pain and suspicion of tennis leg, a soft tissue ultrasound is a simple tool to confirm the diagnosis. However, in the case of negative sonographic findings, we recommend trying a different positional approach to examine the calf muscles by ultrasound before the diagnosis of tennis leg can be ruled out.

## Introduction

Rupture of the distal musculotendinous junction at the medial head of the gastrocnemius muscle is known as "tennis leg" [1,2]. The occurrence of tennis leg is relatively common in athletes who perform sudden acceleration and deceleration maneuvers. The classic clinical manifestation of tennis leg is that of a middle-aged person who complains of acute sports-related pain in the middle portion of the calf muscle associated with a snapping sensation [3]. Imaging tools such as computed tomography (CT), magnetic resonance imaging (MRI) and ultrasound (US) can be used for the diagnosis of tennis leg. Presently, US is most economical and has been used as the primary imaging technique for evaluating patients suffering from tennis leg and other muscle ruptures [1,4].

**Figure 1:**
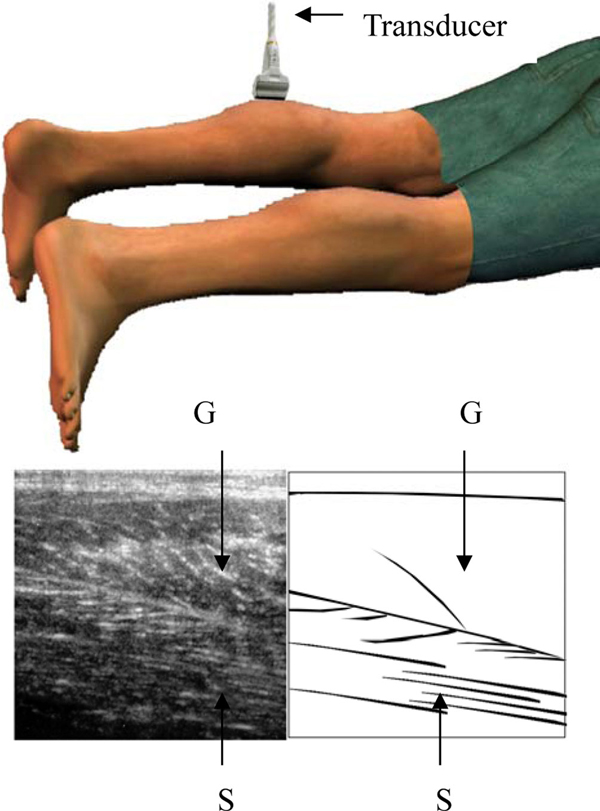
**Longitudinal US images of the medial head of the gastrocnemius muscle (G) and soleus (S) muscle**. The patient was examined in the prone position.

When using US to examine patients suspected of having calf muscle strains, patients are usually placed in the prone position for better viewing of the longitudinal and transverse muscle planes (Figure 1) [1]. Under US, rupture of the medial head of the gastrocnemius muscle can be observed as partial discontinuity of the muscle fibers or as a hyperechoic fluid collection between the gastrocnemius and soleus muscles [1,2]. We present a case of a middle-aged man with sudden onset of left calf muscle pain during rigorous steep mountain climbing. Conventional US examination in the prone position revealed normal sonographic findings. It was not until the third US examination in which a different approach was used to examine the calf muscles that a region of anechoic fluid accumulation was observed between the left gastrocnemius and soleus muscles.

## Case presentation

A 60-year-old man in good health visited our rehabilitation clinic complaining of left calf pain. He visited our clinic 10 days after the sudden onset of pain at the left medial aspect of the posterior calf during rigorous steep mountain climbing. In his words, he felt that the onset of left calf pain was like "being hit by a 100-ton train". Under the impression of the possible rupture of the left medial head of the gastrocnemius muscle, US examination was prescribed.

With the patient in the prone position, US examination was performed by a clinician who was well trained in using soft tissue ultrasound. The SONOS 4500 (Philips Medical Systems, Andover, MA, USA) US machine and S12 5-12 MHz real-time linear-array transducer (Philips Medical Systems) were used to examine the patient. After careful examination, bilateral symmetrical sonographic findings of the calf muscles were noted without evidence of muscle ruptures. Roentgenogram of the left lower limb did not reveal any evidence of bony fractures.

The patient returned to the clinic one week later complaining that the pain in his left calf area persisted and could be further aggravated by tiptoeing and weight bearing maneuvers. Again, US examination in the prone position did not reveal any abnormal sonographic findings.

**Figure 2:**
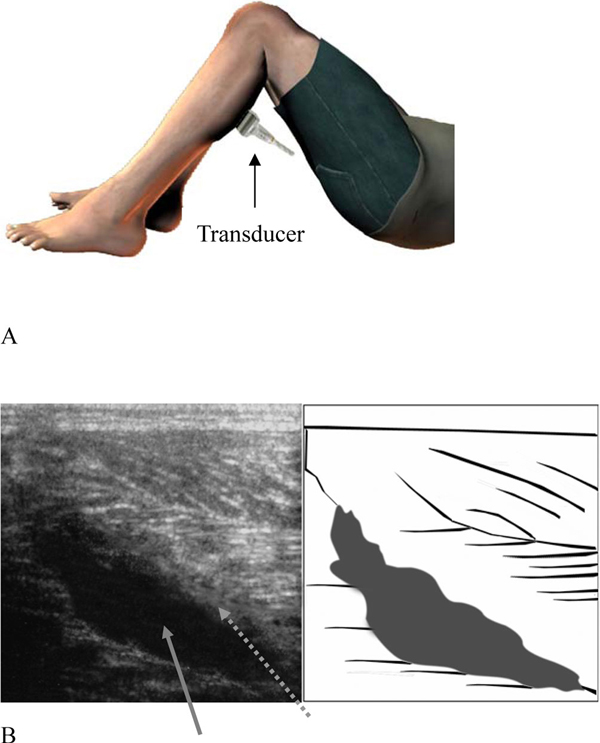
****(A)** The patient was positioned in the supine position with the knee flexed at 90 degrees**. The transducer was now pointing upwards to examine the calf muscles. **(B)** Longitudinal US image examined approximately 17 days after the initial injury revealed a well-defined anechoic fluid collection (grey arrow) site and some hypoechoic areas (dotted grey arrow).

**Figure 3:**
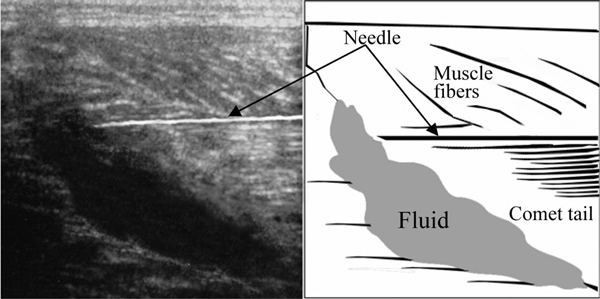
**US guidance of needle insertion for fluid aspiration**.

After two normal sonographic findings in the prone position, the examiner tried a different approach. The patient was placed in the supine position with his knees flexed at 90 degrees (Figure 2A). The transducer was then placed pointing upwards to examine the muscles. An area of well-defined anechoic fluid collection with hypoechoic surroundings was noted (see Figure 2B). Under US guidance, a 21-gauge needle was inserted into the fluid collection area and 15 ml of serosanguinous fluid was aspirated (Figure 3). Dramatic pain relief was noted after aspiration. An elastic stocking was applied to his left calf area after aspiration and follow-up two weeks later did not reveal further fluid accumulations.

## Discussion

Tennis leg is a relatively common clinical condition in athletes [1-3]. A sudden onset of pain is felt in the calf, and patients often experience an audible or palpable "pop" in the medial aspect of the posterior calf [1]. Some patients also feel as if someone has kicked the back of their legs [1]. Patients are usually injured during active plantar flexion of the foot and with simultaneous extension of the knee, which implies active contraction and passive stretching of the gastrocnemius muscle [5], and this seems to be the cause of gastrocnemius muscle rupture in our patient. Our patient experienced sudden onset of severe pain in the left calf area during rigorous steep mountain climbing in which active contraction and passive stretching of the gastrocnemius muscle was believed to be actively involved.

Through scrupulous physical examination, the diagnosis of tennis leg can be easily confirmed. There is often a palpable defect in the medial belly of the gastrocnemius muscle just above the musculotendinous junction. Patients are frequently not able to perform a tip-toeing maneuver on the affected side and experience decreased power upon plantar flexion [1]. US is an effective tool to confirm the diagnosis. In fact, US is useful not only in the initial diagnostic stage, it is also an effective tool to monitor the treatment effectiveness and reparative processes related to tennis leg [1,3,6].

Surprisingly, the usual hyperechoic fluid accumulation noted during acute rupture of the gastrocnemius muscle was not observed by US in our patient in the prone position. Usually, a longitudinal US image of the calf area will reveal a hyperechoic fluid collection between the medial head of the gastrocnemius and soleus muscles during the acute stages of gastrocnemius muscle rupture. The hyperechoic fluid collection represents fresh blood [1]. The reasons that fluid accumulation was not observed by US in our patient in the prone position may be due to:

1. Fluid or blood being dispersed in the lower limb compartments.

2. The degree of muscle tear was not severe enough at the initial stage to observe the partial discontinuity of the muscle fibers [1].

3. The initial blood volume may have been interposed between the medial head of the gastrocnemius and soleus muscles and this may have been mistakenly interpreted as normal fibrous tissues [1,3,7].

With the patient in the supine position and with the knee flexed at 90 degrees, gravity may assist in accumulating all the fluid into one place, which can assist in the viewing of the fluid accumulation at the lesion site using US. Although we have reported only one case, this study may offer the crucial information that when rupture of the gastrocnemius is suspected, a different US examination approach can be applied if the conventional prone position does not reveal any evidence of muscle tear and fluid accumulation.

The treatment of tennis leg is usually conservative and healing of muscle rupture will occur gradually over a period of three to 16 weeks. The US guided needle fluid aspiration performed in this case report is not a routine treatment strategy for tennis leg. Based on the sonographic images gathered, the ruptured muscle was believed to be undergoing reparative processes. The reparative processes [1] were clearly observed under US as hypoechoic areas surrounding the fluid collection site (Figure 2B). We performed fluid aspiration at the patient's request as the bulging painful sensation of his left calf area affected his daily walking routines.

## Conclusion

Loss of muscle fiber continuity and the occurrence of bloody fluid accumulation can be readily observed using US in the prone position in most patients suffering from tennis leg. Although we have reported only one case report, we recommend trying a different positional approach in US examination in patients suspected of having tennis leg when the conventional prone position does not reveal any sonographic evidence of muscle tear.

## Consent

Written informed consent was obtained from the patient for publication of this case report and accompanying images. A copy of the written consent is available for review by the Editor-in-Chief of this journal.

## Competing interests

The authors declare that they have no competing interests.

## Authors' contributions

CC performed the ultrasound examinations and wrote the initial draft of the manuscript. ST was a major contributor in the writing of the manuscript. C-CH was a major contributor in the reading of the sonographic images. RC was a major contributor in the revision of this manuscript. RH was a major contributor in the literature review of this manuscript. WC contributed to the final correction of this manuscript. MC performed all the computer graphic drawings as observed in the figures. All authors read and approved the final manuscript.

## References

[B1] Kwak HS Han YM Lee SY Kim KN Chung GH Diagnosis and follow-up US evaluation of ruptures of the medial head of the gastrocnemius ("tennis leg") Korean J Radiol 2006 7 193 198 10.3348/kjr.2006.7.3.193 16969049 PMC2667601

[B2] Bianchi S Martinoli C Abdelwahab IF Derchi LE Damiani S Sonographic evaluation of tears of the gastrocnemius medial head ("tennis leg") J Ultrasound Med 1998 17 157 162 9514167 10.7863/jum.1998.17.3.157

[B3] Kwak HS Lee KB Han YM Ruptures of the medial head of the gastrocnemius ("tennis leg"): clinical outcome and compression effect Clin Imaging 2006 30 48 53 10.1016/j.clinimag.2005.07.004 16377485

[B4] Delgado GJ Chung CB Lektrakul N Azocar P Botte MJ Coria D Bosch E Resnick D Tennis leg: clinical US study of 141 patients and anatomic investigation of four cadavers with MR imaging and US Radiology 2002 224 112 119 10.1148/radiol.2241011067 12091669

[B5] Miller WA Rupture of the musculotendinous juncture of the medial head of the gastrocnemius muscle Am J Sports Med 1977 5 191 193 10.1177/036354657700500505 907032

[B6] Takebayashi S Takasawa H Banzai Y Miki H Sasaki R Itoh Y Matsubara S Sonographic findings in muscle strain injury: clinical and MR imaging correlation J Ultrasound Med 1995 14 899 905 8583525 10.7863/jum.1995.14.12.899

[B7] Aspelin P Ekberg O Thorsson O Wilhelmsson M Westlin N Ultrasound examination of soft tissue injury of the lower limb in athletes Am J Sports Med 1992 20 601 603 10.1177/036354659202000519 1443331

